# Prediction of clinical outcomes of advanced cutaneous squamous cell carcinoma to PD-1 inhibition directly from histopathology slides using inferred transcriptomics

**DOI:** 10.3389/fimmu.2026.1822422

**Published:** 2026-05-13

**Authors:** Bohdana Chayen, Gal Dinstag, Omer Tirosh, Leon Gugel, Tuvik Beker, Tzivia Gottlieb, Anna Elia, Eli Pikarsky, Michal Lotem, Mordechai Avner, Jonathan E. Cohen, Ranit Aharonov, Aron Popovtzer, Johnathan Arnon

**Affiliations:** 1 Sharett Institute of Oncology, Hadassah-Hebrew University Medical Center, Jerusalem, Israel; 2 Pangea Biomed Ltd., Tel-Aviv, Israel; 3 Department of Pathology, Hadassah-Hebrew University Medical Center, Jerusalem, Israel; 4 The Lautenberg Center for Immunology, IMRIC, the Hebrew University of Jerusalem, Jerusalem, Israel; 5 The Hadassah Cancer Research Institute, Hadassah Hebrew University Medical Center, Jerusalem, Israel; 6 Faculty of Medicine, Hebrew University of Jerusalem, Jerusalem, Israel

**Keywords:** biomarker, cemiplimab, cutaneous squamous cell carcinoma, digital pathology, immunotherapy, transcriptomics

## Abstract

**Introduction:**

Metastatic or locally advanced cutaneous squamous cell carcinoma (cSCC) that is not amenable to local therapy is treated with programmed death-1 (PD-1) inhibitors. Although response rates are relatively high, there are no validated predictive biomarkers to guide treatment. As a result, a subset of patients — particularly frail and elderly patients which can be treated with local palliative therapy — are exposed to immune-related adverse events without clinical benefit. Here, we present a retrospective evaluation of ENLIGHT-DP, a novel digital pathology biomarker which predicts response to PD-1 inhibition in advanced cSCC directly from histopathology slides using inferred transcriptomics.

**Methods:**

We scanned high-resolution hematoxylin and eosin (H&E) slides from pretreatment tumor samples of 38 patients with advanced cSCC treated with cemiplimab and retrospectively generated an individualized prediction score using the ENLIGHT-DP pipeline in a two-step process: (i) inference of mRNA expression profiles directly from H&E slides using the DeepPT deep-learning algorithm, and (ii) integration of these inferred transcriptomes into ENLIGHT, a transcriptomics-based precision oncology platform that predicts therapeutic response. We unblinded clinical outcomes and assessed the predictive performance of ENLIGHT-DP.

**Results:**

The cohort consisted primarily of frail, elderly patients (median age 81 years), with 18 patients having an ECOG performance status ≥2. Using a binary threshold for classification, ENLIGHT-DP significantly predicted response to cemiplimab, demonstrating a positive predictive value of 84.2% and an odds ratio (OR) of 4.8 (95% CI: 1.1–22.1), along with significant stratification for progression-free survival with HR = 0.22 (95% CI: 0.05–0.95, p = 0.023) and outperforming performance status, age and site of cancer. Comparative analyses of inferred immune-related transcriptomic signatures revealed significant differences between cSCC and head and neck squamous cell carcinoma, underscoring distinct tumor immunobiology.

**Conclusion:**

This exploratory study introduces ENLIGHT-DP as a digital pathology biomarker shown to significantly predict clinical outcomes in patients with cSCC treated with PD-1 inhibitors.

## Background

Cutaneous squamous cell carcinoma (cSCC) is the second most common skin cancer worldwide, representing a major public health burden (1). While most patients are diagnosed with localized disease, a subset of patients —estimated at 3–5% of cases—develop locally advanced or metastatic disease not amenable to curative treatment by surgical excision or radiotherapy (1). The introduction of immune checkpoint inhibitors (ICI) has revolutionized the therapeutic landscape of advanced cSCC. Programmed death-1 (PD-1) inhibitors, namely Cemiplimab and Pembrolizumab, provide durable responses in nearly 50% of patients with advanced cSCC, circumventing aggressive therapies for locally advanced tumors and prolonging overall survival (OS) in metastatic disease (2, 3). Despite these advances, a substantial proportion of patients fail to respond or eventually develop resistance to ICI, resulting in a median progression-free survival (PFS) of 12 months or less (3, 4). Furthermore, high-grade immune-related adverse events (irAE) - which occur in almost 30% of cases - remain a major concern. To date, no clinically validated biomarkers exist to support treatment decision making in advanced cSCC. As many patients with advanced cSCC are older, frail or have multiple co-morbidities, and present with locally advanced disease, it remains unclear which patients would be better treated with local palliative therapy rather than ICI (5–7). Therefore, there is an urgent need for accurate and easily applicable biomarkers to guide treatment decision making in advanced cSCC.

PD-L1 expression has been explored as a potential biomarker in cSCC treated with ICI, yet its predictive value remains inconsistent, likely due to enrichment of PD-L1 expression in advanced cSCC tumors and high response to ICI across all PD-L1 expression levels (9). cSCC is shaped by chronic ultraviolet (UV)-induced DNA damage and is highly immunogenic with a rich immune-cell microenvironment and high tumor mutational burden (TMB) (8, 9). Hence, immune-based biomarkers are potential candidates to predict outcomes of ICI in cCSCC. Immune gene expression signatures, immune-cell phenotyping and TMB, have shown promising results in retrospective analyses, but are not yet validated for clinical use and require extensive molecular testing or additional immunohistochemistry staining which are resource-intensive and not routinely done in advanced cSCC (10).

**Figure 1:**
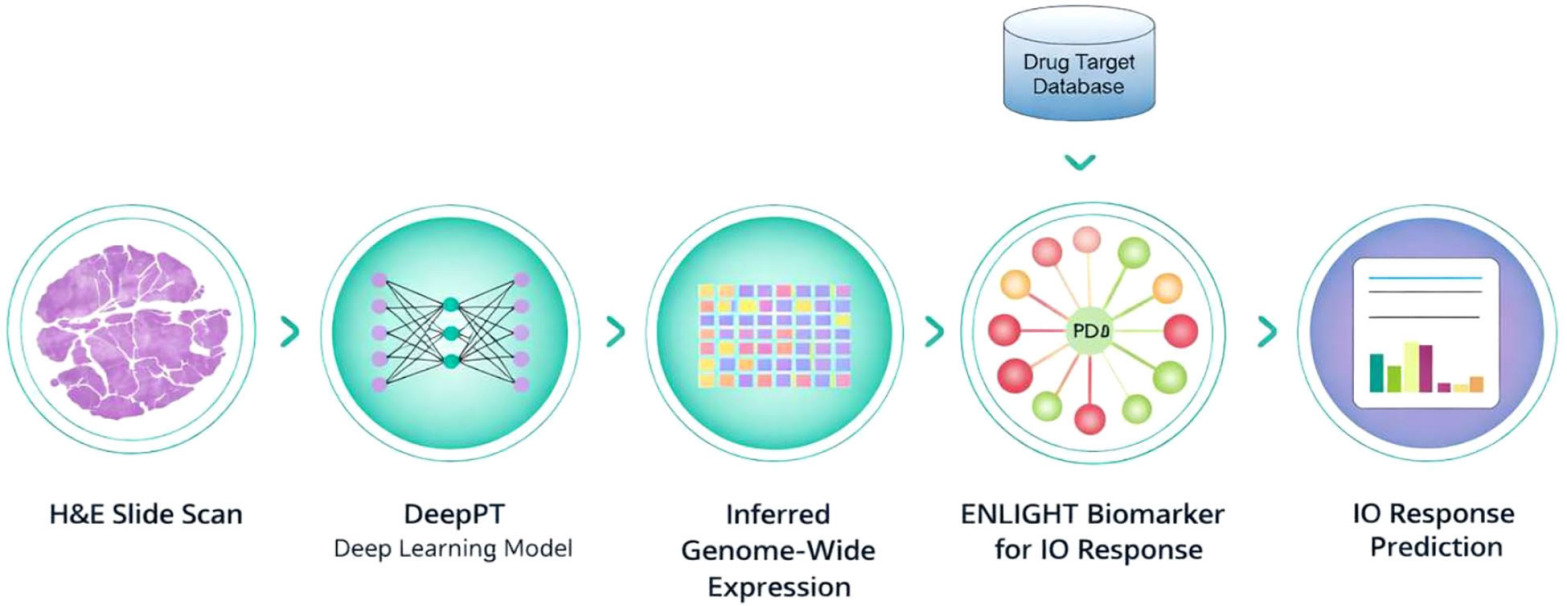
The ENLIGHT-DP pipeline ENLIGHT-DP generates an individualized prediction scores through a two-step process: (i) inference of genome-wide RNA expression profiles directly from hematoxylin-and eosins (H&E) scanned slides using the DeepPT deep-learning algorithm, and (ii) integration of these inferred transcriptomes into ENLIGHT, a transcriptomics-based precision oncology platform that predicts therapeutic response to immunotherapy and other cancer therapies.

ENLIGHT-DP, developed by Pangea Biomed, is a digital-pathology biomarker that predicts individual responses to ICI and targeted therapies directly from scanned histopathology slides by inferred transcriptomics as previously described (11, 12). Briefly, the ENLIGHT-DP pipeline consists of: (i) ENLIGHT, a computational platform derived from the analysis of functional interactions between genes across the human genome. These interactions are used to construct drug-specific gene networks, are predictive of therapeutic response to targeted therapies and immunotherapy. The gene networks are typically composed of 10 genes whose activation levels are measured by mRNA expression profiling through next-generation sequencing (NGS) or microarrays (12) (ii) DeepPT is a complementary deep-learning artificial intelligence model which infers genome-wide mRNA expression from standard hematoxylin-and eosins (H&E) histopathology scanned slides. The integration of DeepPT as an input source for ENLIGHT - termed ENLIGHT-DP- allows prediction of response to several targeted therapies and ICI with high accuracy directly from histopathology slides scans by inferred transcriptomics (11) (**Figure 1***)*. This model was originally trained on 16 large cancer cohorts, including head and neck, cervical, and lung squamous cell carcinomas, demonstrating a strong concordance between expression measured from RNA and inferred from digital pathology, across more than 18,000 genes, resulting in accurate prediction of clinical outcomes across these cohorts. Importantly, the model accurately predicted treatment responses in five independent patient cohorts, supporting its generalizability to other cancer types and treatments and enabling its implementation in clinical scenarios with limited datasets, while maintaining improved predictive accuracy (11). This tool enables stratification of clinical outcomes from highly available histopathology slides while circumventing costly and resource-consuming transcriptomic analyses and thus, removing the primary barrier to transcriptomic-based personalized treatment selection.

In subsequent real-world validation cohorts, we recently demonstrated that ENLIGHT-DP significantly predicts response and PFS in patients with lung adenocarcinoma and head and neck squamous cell carcinoma (HNSCC) as well as pan-cancer cohorts treated with first-line ICI, outperforming PDL-1 expression and TMB (13, 14, 16). Here, we present a retrospective exploratory analysis for the application of ENLIGHT-DP in patients with advanced cSCC treated with first-line PD-1 inhibitors in a real-world setting. We further compare the inferred transcriptomic signature of cSCC to our previously published HNSCC cohort treated with first line PD-1 inhibitors and provide insights on the different immunobiology and distinctive responses to PD-1 inhibition between the two tumors derived directly from H&E slides.

## Methods

### Study design

This is a retrospective, single-center, real-world study conducted at Hadassah–Hebrew University Medical Center (HMC) in collaboration with Pangea Biomed. Eligible cases were identified from the institutional oncology and pathology databases. High-resolution H&E slides from pre-treatment tumor biopsies were digitally scanned and analyzed using the ENLIGHT-DP pipeline to generate individualized prediction scores for PD-1 inhibitors. Clinical outcomes were then unblinded, and the predictive value of ENLIGHT-DP was calculated and compared to relevant clinical characteristics namely - performance status, age, site and origin of disease (4, 7). Inferred transcriptomic signatures of this cohort and a recently published HNSCC cohort were compared to elucidate the underlying mechanisms of different responses to PD-1 inhibitors.

### Participants

We included patients who were diagnosed with advanced cSCC not amenable to curative treatment, as a result of the extent of local disease, anticipated surgical or radiotherapy complications, regional or distant metastasis. We included only patients who had available archived H&E histopathology slides from pre-treatment biopsies, were treated with first line PD-1 inhibitors for advanced disease and had sufficient follow up of at least 6 months for surviving patients or earlier in case of death. In cases in which multiple biopsies were available from the same patient e.g., from diagnostic and recurrent disease biopsies, we selected the sample more recent to time of treatment initiation. We included patients with prior cancer diagnosis, only if this cancer was ruled out before Cemiplimab treatment for advanced cSCC was initiated.

### Data collection and clinical outcomes

Clinical, demographic, and pathological data were retrieved from electronic medical records, including age, gender, comorbidities, performance status by Eastern Cooperative Oncology Group (ECOG), location and burden of disease, and prior therapies for local disease. The primary clinical outcomes were best overall response (BOR), assessed by institutional radiologists according to iRECIST 1.1 criteria and PFS, defined as time from PD-1 inhibitor initiation to progression or death. Other clinical outcomes included OS defined as time from treatment initiation to death from any cause, with surviving patients censored at the last follow-up; duration of response (DOR), defined at time from treatment start to progression or death for the responding patients and irAE assessed by treating physician.

### ENLIGHT-DP algorithm and computation of the ENLIGHT-DP matching score

Pre-treatment H&E slides were scanned at 20× magnification (<0.5 µm per pixel) and analyzed through the ENLIGHT-DP pipeline. For each case, an ENLIGHT-DP matching score (EMS) was calculated via three sequential stages: (i) Transcriptome inference: the indication-specific DeepPT model predicted whole-transcriptome gene-expression profiles from digitized H&E slides (12). In this study, we used the HNSCC DeepPT model to predict transcriptomics, since DeepPT has no data to train an cSCC model exists, and to have an appropriate downstream comparison to HNSCC as will be illustrated later (13); (ii) Gene activation profiling: predicted expression levels were transformed into categorical activation states (over-, under-, or normally expressed) for each gene based on percentile distribution across the study cohort, as previously described (11); (iii) Response modelling: gene-activation patterns were integrated with the ENLIGHT response network for PD-1 inhibition to generate an individualized ENLIGHT Matching Score (EMS) (**Figure 1**). The EMS ranges from 0 to 1, with higher values indicating greater predicted sensitivity to PD-1 inhibitors. To support clinical decision-making, the EMS was primarily applied as a binary classifier, using the median EMS to stratify patients into EMS-matched versus EMS-unmatched groups. In addition, the biomarker was evaluated using thresholds previously established for HNSCC cohorts, and as a continuous score.

### Statistical analysis

Descriptive statistics were reported as medians with range or 95% CIs for continuous variables, and as percentages for categorical variables. Response rates were compared between EMS-matched and unmatched groups and between clinical characteristics and reported as odds ratios (OR) using Mann Whitney U or one-sided proportion tests. Predictive value for BOR was estimated using the area under the receiver operating characteristic curve (ROC-AUC).PFS and OS were assessed using the Kaplan–Meier method, and group comparisons were conducted via the log-rank test. Hazard ratios (HRs) and 95% CIs were calculated using Cox proportional-hazards models. Two-sided *p* values <0.05 were considered statistically significant. All statistical analyses were performed using R software (version 4.3.3).

## Results

### Patient characteristics

Between January 2020 and December 2024, a total of 40 patients were diagnosed with advanced cSCC and treated with first-line Cemiplimab. Of these, 38 patients had available pre-treatment H&E histopathology slides, adequate follow-up data and were included in the study. The median age of diagnosis was 81 years (range 57–100) and most patients presented with multiple comorbidities or prior cancer diagnosis. Sixteen (42.1%) patients had an ECOG of 0–1, and 18 patients (47.4%) had an ECOG score ≥2, reflecting a predominantly frail and elderly representative of the advanced cSCC population. Two patients (5.3%) had a history of immunosuppression resulting from prior lymphoma and sarcoidosis treatment. Of the 38 patients, 32 (84.2%) had previously undergone surgery or radiotherapy with curative intent but subsequently experienced disease progression not amenable for local therapy. Further patient characteristics are summarized in **Table 1**.

**Table 1:** Clinical characteristics of 38 patients with cutaneous squamous cell carcinoma (CSCC) treated with Cemiplimab for advanced or metastatic disease.

Patient characteristic	N=38
Age at diagnosis	
Median (Range) – year	81 (range 57-100)
>65 yr -no (%)	33 (86.8%)
Gender – no (%)	
Female	13 (34 %)
Male	25 (66%)
Ethnic backgrounds	
Arab	6 (15.8%)
Ashkenazi Jewish	20 (52.6%)
Non-Ashkenazi Jewish	12 (31.6%)
ECOG performance status score – no. (%)	
0	4 (10.5%)
1	12 (31.6%)
≥ 2	18 (47.4%)
Unknown	4 (10.5%)
Most frequent comorbidities:	
Diabetes	14 (36.8%)
Hypertension	11 (28.9%)
Ischemic Heart Disease	7 (18.4%)
Chronic Renal Failure	5 (13.2%)
Atrial Fibrillation	5 (13.2%)
Other cancer-related diagnoses:	
Basel Cell Carcinoma	5 (13.2%)
Breast	2 (5.3%)
Melanoma	2 (5.3%)
Lung, Breast, Cervix, Bladder, Vocal Cord (1 each)	5 (13.2%)
Primary Tumor Location	
Head and neck	28 (73.7%)
Chest and trunk	4 (10.5%)
Limbs	6 (15.8%)
Burden of disease	
Locally advanced	36 (94.7%)
Metastatic	2 (5.3%)
Previous Treatment for CSCC	
No previous treatment	6 (15.8%)
Previous treatment:	32 (84.2%)
- Platinum-based chemotherapy	- 5 (13.2%)
- Radiotherapy	- 24 (63.2%)
- Surgery	- 25 (65.8%)

### Treatment outcomes

At time of data cut-off (October 2025) with a median follow-up time of 23.0 months (95% CI 20.0-30.0), none of the patients were still receiving Cemiplimab and 28 patients (73.68%) were still alive. The BOR was 68.4%, comprising 19 patients (50%) who achieved a complete response (CR) and 7 patients (18.4%) who achieved a partial response (PR). Median DOR was 21.0 months (95% CI: 14.0–33.7 months). The median PFS was 13.5 months (95% CI: 7.0–21.5 months), and the median OS could not be determined. In 17 cases (44.7%) treatment was discontinued due to progression (n=15), irAE (n=1) or death (n=1), while in the remaining 21 cases (55.3%) therapy was stopped due to sustained response and shared patient-clinician decision. Notably, only one patient in this cohort died from a cancer-specific cause, whereas the remaining 9 deaths were attributable to age-related causes and other comorbidities.

There was one documented case of grade 3 irAE involving autoimmune hepatitis. In this case cemiplimab was withheld and systemic corticosteroids were administered with resolution. In addition, there were 13 documented cases (34.2%) of grade 1–2 irAE, none of which required treatment discontinuation, including skin toxicity (n=2), hyperglycemia (n=1), creatinine elevation (n=4), and fatigue (n=4) as well as incidental findings of asymptomatic mild pericarditis (n=1) and pneumonitis (n=1) diagnosed after treatment had already been discontinued.

### ENLIGHT-DP is predictive of response and progression-free survival

**Figure 2:**
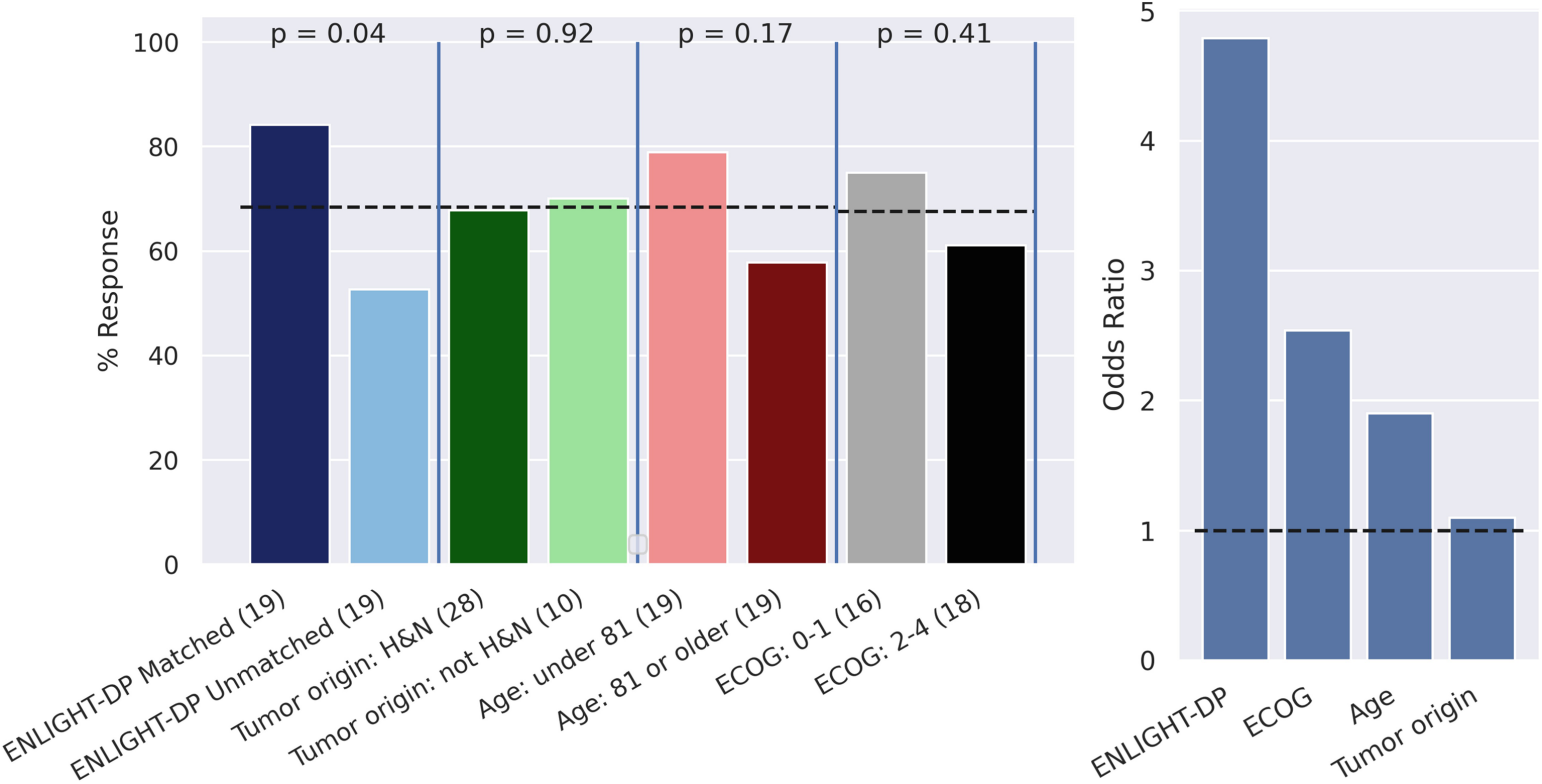
Response to immune checkpoint inhibition according to ENLIGHT-DP and clinical characteristics. Left: Response rate among patients with cutaneous squamous cell carcinoma (cSCC) treated with Cempilimab according to different patient groups - ENLIGHT-DP (matched vs. unmatched), ECOG (0–1 vs. ≥2) age (under 81 vs. 81 years or older) and tumor origin (head and neck vs. other). P-value for prediction of response according to each parameter calculated using Man Whitney U test. The dashed line represents the baseline response rate. Right: Odds Ratio (OR) for response for ENLIGHT-DP (OR 4.8 95% CI: 1.1–22.1), ECOG (OR 2.5; 95% CI 0.4-8.3), age (OR 1.9; 95% CI 0.6-10.7) and tumor origin (OR 1.1; 95% CI 0.2-5.3). Dashed line denotes an OR of 1, corresponding to a random classifier.

**Figure 3:**
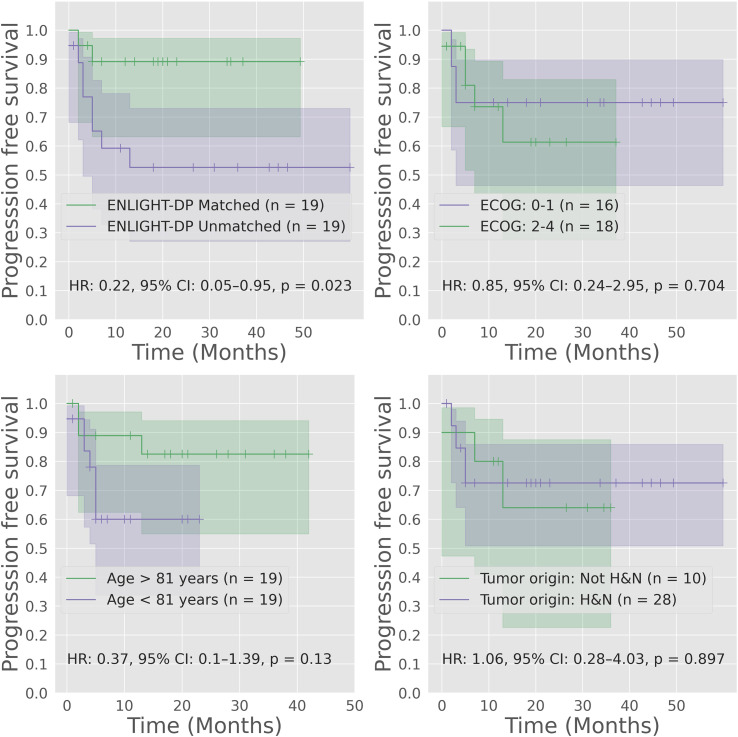
Progression-free survival of immune checkpoint inhibition according to ENLIGHT-DP and clinical characteristics. Kaplan Meir (KM) plots for progression-free survival (PFS) stratifying patients with cutaneous squamous cell carcinoma (cSCC) treated with Cempilimab stratified according to ENLIGHT-DP (upper-left, matched vs. unmatched), ECOG (upper-right, 0–1 vs. ≥2) age (bottom left, under 81 vs. 81 years or older) and tumor site (bottom right, head and neck vs other). Hazard ratio (HR) calculated using cox-regression.

**Figure 4:**
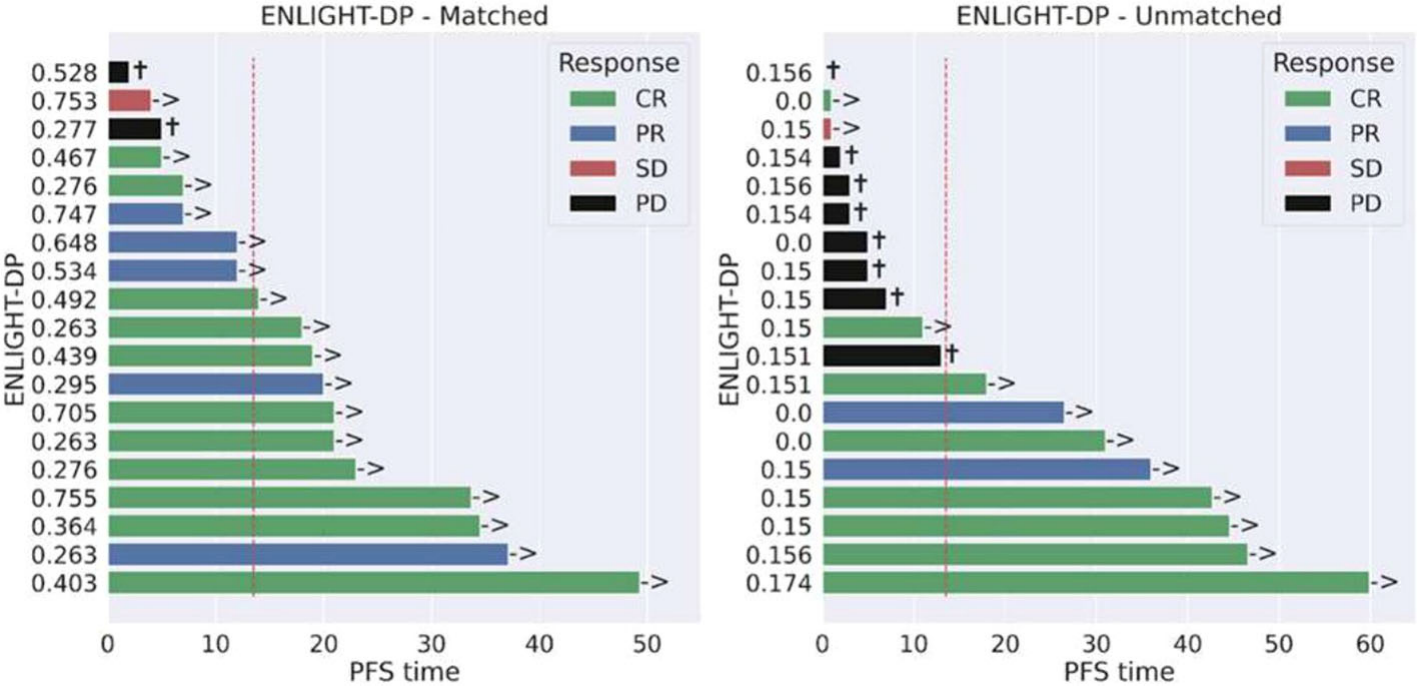
Individual outcomes of immune checkpoint inhibition according to ENLIGHT-DP Individual progression free survival (PFS) and best overall response distribution among ENLIGHT-DP matched patients (left) and unmatched patients (right) at time of data cut-off. Y-axis denotes the individual ENLIGHT-DP matching score (EMS). The red dashed line denotes the median PFS of the entire population (13.5 months).

To support clinical decision making, we focussed on the predefined median EMS score as a threshold for classification into EMS-matched and EMS-unmatched groups. Using this classifier, ENLIGHT-DP had an odds ratio (OR) for response of 4.8 (95% CI: 1.1–22.1) a positive predictive value (PPV) of 84.2%, representing a 23.1% lift over the baseline response rate of 68.4%, sensitivity of 61.5%, negative predictive value (NPV) of 47.3% and specificity of 75.0%. ENLIGHT-DP is more predictive of response than ECOG status (OR 2.5; 95% CI 0.4-8.3), age (OR 1.9; 95% CI 0.6-10.7) and tumor origin (OR 1.1; 95% CI 0.2-5.3) (**Figure 2**). ENLIGHT-DP showed a significant stratification in PFS between EMS-matched and EMS-unmatched patients with HR: 0.22 (95% CI: 0.05–0.95, p = 0.023) while ECOG (0–1 vs. ≥2) tumor site (head and neck vs. other) and age were not significant in stratifying patients (**Figure 3**). Individual outcomes according to EMS are provided in **Figure 4**. Notably, in 4 of 38 cases (10.5%), analysis was carried out on biopsies taken more than one year before systemic therapy was initiated. Nevertheless, inclusion of these cases did not influence the results. Furthermore, employing binary cutoffs utilized in our previous HNSCC cohorts (14) yielded comparable results for prediction of response and PFS. Analysis of EMS as a continuous score for prediction of BOR in response to PD-1 inhibition resulted in a ROC AUC of 0.61.

### Comparison of immune signatures of cSCC and HNSCC

**Figure 5:**
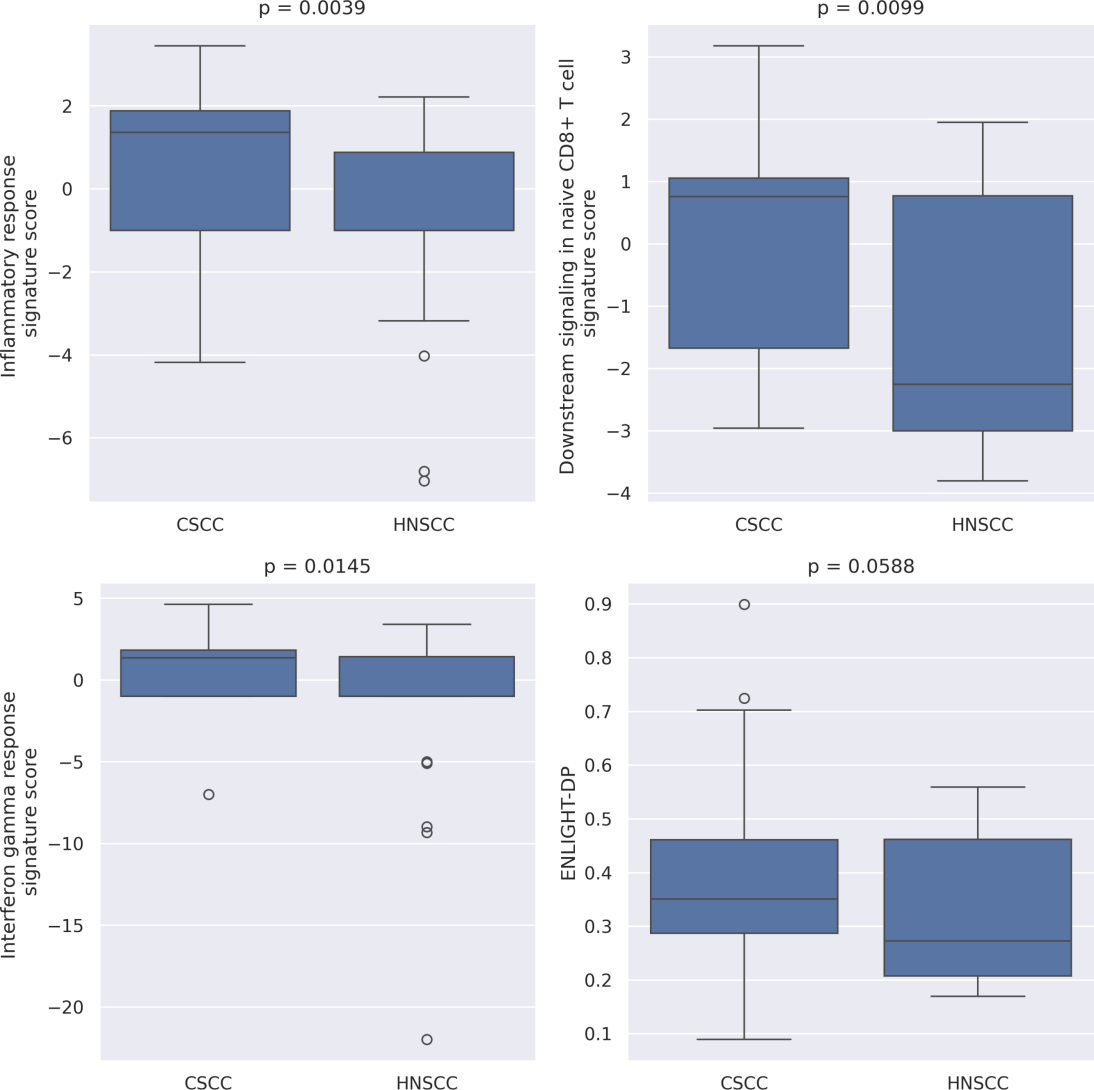
Inferred transcriptomic analysis of three immune-based signatures and total ENLIGHT-DP score, comparing cutaneous squamous cell carcinoma (cSCC, n = 38) and head and neck squamous cell carcinoma (HNSCC, n = 25) cohorts, cSCC exhibits higher immune signature scores across all three immune-based signatures and higher general ENLIGHT-DP scores compared with HNSCC patients. P-value was calculated using Mann-Whitney U test.

We analyzed three different inferred immune-based signatures which comprise ENLIGHT-DP - inflammatory response, interferon-γ (IFNγ) and CD8 T-cell downstream signaling - and compared this to a previous HNSCC cohort. All three immune-based signature scores showed significantly higher values in the cSCC cohort compared to HNSCC (**Figure 5**). We also observed generally higher ENLIGHT-DP scores when comparing the entire cSCC and HNSCC cohorts, though this difference was not significant (p = 0.059). This analysis further testifies to the elevated immunological predisposition of cSCC compared to HNSCC, underscoring its higher response rates for PD-1 inhibition and demonstrating that broader biological meaning can be drawn from the H&E slides by inferred transcriptomics.

## Discussion

This cohort introduces ENLIGHT-DP as a digital-pathology biomarker that significantly predicted response and PFS to ICI treatment in advanced cSCC by inferred transcriptomics. Using the clinically applicable binary threshold for classification, ENLIGHT-DP displays promising biomarker characteristics: OR 4.8 (p = 0.04) for response and a significant separation of PFS between EMS-matched and EMS-unmatched patients with HR 0.21 (p = 0.02), outperforming clinical characteristics (ECOG, age, origin of tumor). The high PPV of 84.2% enables the identification of patients with locally advanced disease, representing the majority of cSCC patients, who are likely to benefit from ICI, rather than local therapies in the form of repeated radiotherapy or aggressive surgery. These findings underscore the potential of integrating this digital-pathology biomarker for personalized transcriptomic-based treatment without reliance on resource-intensive genomic analyses which are unavailable in routine cSCC work-up. This is particularly relevant given the lack of clinically validated biomarkers for ICI treatment in cSCC.

Due to its retrospective single center design and limited sample size, this study is exploratory in nature, aiming to evaluate the potential clinical utility of EMS in advanced cSCC and adding to previously published validation cohorts in breast cancer, HNSCC, metastatic NSCLC as well as pan-cancer cohorts (13–15) demonstrating the robustness of the ENLIGHT-DP approach for predicting outcomes of ICI therapy across distinct populations and tumor types. Further studies should aim to validate ENLIGHT-DP in larger, multi-center prospective cohorts and confirm its generalizability across different clinical settings and populations including immunosuppressed patients and in the neoadjuvant ICI treatment in cSCC to enable its translation into clinical application. In this Cohort ENLIGHT-DP did not stratify patients in regard to OS, as majority of patients were still alive at time of data cut-off. Of note, survival is not commonly regarded as a primary clinical outcome of advanced cSCC given that many patients are frail and older. Consistent with this, only one of the ten deaths observed in this cohort was attributed to progressive disease, while the remaining nine deaths were due to non–cancer-related causes.

### Comparison to other transcriptomic and digital pathology biomarkers

Deep-learning digital pathology tools in cSCC were previously utilized mainly to predict progression and potential of metastatic spread from localized cSCC lesions (16, 17). To our knowledge this is the first use of a digital pathology biomarker utilized to predict treatment outcomes and stratify patient populations in cSCC treated with ICI. Further implementation of ENLIGHT-DP in the local curative setting would also allow for more in-depth analysis of the algorithm’s ability to distinguish between rapid and slow metastatic progression, as well as stratify patients which are candidates for neoadjuvant ICI treatment. Several other transcriptomic-based tools for prediction of ICI treatment response in advanced cSCC show significant patient stratification including, tumor tissue-derived (e.g., squamous cell stemness, differentiation, growth, and inflammation and “immunse-score”) as well as liquid biopsy-based (e.g., sPD-L1, sLAG-3, sTIM-3, sCTLA-4, and cfDNA). Recently, elevated baseline serum levels of IFN-γ were found to be significantly associated with poorer treatment response and shorter PFS (10, 18, 19). While identifying gene expression signatures could provide deeper insights into treatment response and help discover potential therapeutic targets, these tools require extensive genomic analysis of tumors or blood which is not routinely done in cSCC and therefore lack clinical applicability.

In this study, we further demonstrate how ENLIGHT-DP can be used to characterize gene expression signatures through inferred transcriptomics derived directly from H&E-stained slides, highlighting the potential of ENLIGHT-DP as a research tool. The differences observed across three immune-related signatures between the cSCC and HNSCC cohorts provide additional evidence of the distinct immunobiology of these two tumors, despite their shared histological features, and demonstrated that broader biological meaning can be drawn from the H&E slides using the ENLIGHT-DP pipeline.

### Clinical outcomes compared to previously published cohorts

The clinical outcomes in this cohort, namely median PFS of 13.4 and BOR of 68.4% align with previous real-world studies in similar patient cohorts receiving PD-1 inhibitors therapy for advanced cSCC (5, 7). A BOR of 68.4% and a PFS of 8.9 months have been previously reported in elderly and immunocompromised patients treated with PD-1 inhibitors for advanced cSCC, highlighting the clinical efficacy of this therapeutic class in vulnerable populations (20). The relatively higher rate of BOR in our cohort, as well as in other real-world studies, likely results from the non-independent assessment which differs from the centralized assessment implemented in prospective trials (3, 4). The rate of irAEs in our cohort is consistent with findings from other retrospective studies involving elderly and frail patients which reported PD-1 inhibition as generally well-tolerated, with most irAE being mild to moderate (5, 6, 20). Nevertheless, in both prospective and retrospective studies, several cases of death have been attributed to severe irAEs, underscoring the importance of improved patient stratification and of avoiding ICI in patients who are unlikely to benefit, are frail, or have other palliative treatment options.

Interestingly, nearly 50% of responding patients in our cohort stopped treatment after achieving response, regardless of irAE, without experiencing subsequent disease progression. Understanding optimal discontinuation strategies for immunotherapies, particularly in elderly and frail patients, is essential to minimize overtreatment while maintaining clinical benefit (21, 22). Recent evidence supports safe ICI discontinuation after 12 months in cSCC regardless of response or even earlier if CR is achieved (23). However, variations in treatment duration and discontinuation strategies across these studies suggest a need for further investigation into optimal treatment protocols.

### Study limitations

This study offers valuable insights but has several limitations. While the median follow-up time of 32 months is sufficient, the small sample size (n=38) and the retrospective single-center design inherently introduce biases that restrict the generalizability of the findings to broader patient populations. This is relevant in regard to the general cSCC population and more specifically to patients with a history of immunosuppression and metastatic disease, which were underrepresented in this cohort (n=2 each). Nevertheless, the study is representative of the advanced CSCC population, characterized by male predominance (66%), older yet diverse age (range 57-100), a range of ECOG-statuses (with 18 patients having ECOG >1), and varied ethnic backgrounds (Arab:6 Ashkenazi Jewish: 20, and Non-Ashkenazi Jewish: 12). Another major limitation is the lack of PD-L1 immunohistochemistry staining, TMB and genomic data, which limits the comparison of the performance of ENLIGHT-DP in cSCC to other histopathological or genomic-based biomarkers.

## Conclusion

This exploratory study introduces ENLIGHT-DP as a digital pathology biomarker shown to significantly predict clinical outcomes in patients with cSCC treated with PD-1 inhibitors. ENLIGHT-DP addresses a critical unmet need in a field lacking validated predictive biomarkers, with particular relevance to cSCC, and highlights the potential of ENLIGHT-DP to enable personalized treatment stratification without reliance on resource-consuming genomic analyses. Future multicenter and prospective validation studies, comparisons with established biomarkers and evaluation in additional clinical settings will be essential to translate ENLIGHT-DP into clinical practice and ultimately optimize treatment for patients with cSCC.

## Data Availability

The original contributions presented in the study are included in the article/supplementary material. Further inquiries can be directed to the corresponding authors.
